# Borderland Studies

**Published:** 1909-05

**Authors:** 


**Borderland Studies.** By **George M. Gould,** M.D., Author of a Series of Medical Dictionaries, etc. Vol. 2, 12 mo, pp. 311. Illustrated. Philadelphia: P. Blakiston's Son & Co.

This second volume of gathered essays, addresses and lectures by Dr. Gould is a most interesting contribution, full of the delight of the grace of this accomplished writer. There are fourteen chapters and there isn't a dull or commonplace page between the covers.

Especially interesting is The History of the House. The Seven Deadly Sins of Civilisation is a terrifying title. One proceeds to a reading with trepidation and then loses some of the awe which has enwrapped him when he finds these seven deadly sins to be composed of tobacco, coffee and tea, alcohol, sugar, venereal diseases, the modern house and, of course, eye strain. King Arthur's Medicine is an exceptionally interesting and instructive chapter and it is wholly charming. Child Fetiches is quite the best chapter in the book, while the Story and Lessons of an Unknown Hero's Life ought to be read by the whiners and perpetual grouches who give us no surcease of their pestiferous self-sorrow. N. W. W.

